#  Effect of Cholesterol and Equex-STM Addition to an Egg Yolk Extender on Pure Spanish Stallion Cryopreserved Sperm

**DOI:** 10.1155/2013/280143

**Published:** 2013-12-12

**Authors:** Lidia Gil, Iván Galindo-Cardiel, C. Malo, N. González, C. Álvarez

**Affiliations:** ^1^Reproduction and Obstetrics Area, Department of Animal Pathology, Faculty of Veterinary, University of Zaragoza, Spain; ^2^WorldPathol Ltd. Co., Zaragoza, Spain; ^3^Equine Reproduction Center, Torre Abejar, Garrapinillos, Zaragoza, Spain

## Abstract

Cholesterol and Equex-STM are frequently added to different commercial and experimental extenders improving postthawing sperm quality. Doses of 125–150 mM of cholesterol from pig liver and 0.5–0.7% of Equex-STM were evaluated in a standard eggyolk extender (Martin et al., 1979). Six ejaculates per stallion from six pure Spanish stallions (6–8 years old) were collected in Martin's extender (B) and different mixtures of 125 mM-0.5% (I), 125 mM-0.7% (II), 150 mM-0.5% (III), and 150 mM-0.7% (IV) were added to original Martin's extender. Samples were frozen in 0.5 mL straws (100 × 10^6^ spermatozoa) and thawed (21 s., 37°C water bath). After thawing the following parameters were evaluated: viability (V), motility (computer assisted sperm analysis, CASA; % nonprogressive NP; % progressive MP), hipoosmotic swelling test (HOST), acrosome integrity (A), fluorescence test (FL), and resistance test (RT). Sperm quality was significantly affected by stallion (in the parameters V, VI, NP, MP, HOST, A, FL, and RT), extraction (VI, NP, MP, HOST, A, and FL), and the different combinations of Equex-STM-cholesterol (FL). We concluded that 0.5% of Equex-STM mixed with 125 mM of cholesterol has obtained better sperm quality results than those of original Martin's extender, showing a simple and economic improvement of this home-made practical seminal extender.

## 1. Introduction

Artificial insemination remains as one of the most important assisted reproductive technologies. It is a simple, economical and successful method for animal reproduction [1]. Freezing/thawing sperm techniques are a widely used method to manage equine breeds and maintain genetic stallion pool [2]. Different sperm cryopreserving methods have been developed for a lot of species, such as Sea Urchin (*Evechinus chloroticus*) [3], Carp (*Cyprinus carpio*) [4], Pacific oyster (*Crassostrea gigas*) [5], Red Deer (*Cervus elaphus*) [6], Human (*Homo sapiens sapiens*) [7], and practically all domestic and farmer species (reviewed by [8, 9]). Seminal cryopreservation techniques should be performed taking into account physiological individual stallion-depending sperm variation and the specific cooling, freezing, and thawing rates, such as the necessary equipment [10–14].

Frozen/thawed sperm protocols have been described as an improving reproductive tool related to a better breed management and reproductive potential stallion optimization due to its ability to preserve fertile inseminating doses [15, 16]. Cryopreservation extends the availability of sperm for fertilization; however, the fertilizing potential of the frozen-thawed sperm is compromised because of alterations in the structure and physiology of the sperm cell [17–19]. Sperm capacitation-like alterations are present in the motile population and decrease sperm life span, interaction with female tract, and its fertilizing ability [20]. The etiology of such alterations may represent a combination of factors, such as inherited fragility of the sperm cell to withstand the cryopreservation process and the semen dilution. Sperm membrane cholesterol, reactive oxygen species, and seminal plasma have been related as mediators of cryopreservation effects on sperm functions, just as capacitation process [8, 9, 21–23].

Several articles have been carried out to analyze the effect of the cholesterol added as cryoprotective agent, using different techniques such as cyclodextrin incubation, liposomes, or low density lipoproteins, getting different results which will be discussed [24–26]. The addition of Equex-STM has been tested to yield high cryopreservation results, mainly in the dog [27–32], stallion [33], lama (*Lama glama*) [34], boar [35, 36], gazelle [36], rat, and sheep [28, 32, 37, 38]. As for other lauryl sulphate derivatives, the positive effects of these compounds are thought to be brought about by two combined mechanisms: interaction between the egg yolk in the extender and Equex-STM, modifying the protein structure of lipoproteins [35, 39] and improving the stability of plasma membrane lipids [27] by adding cholesterol.

The goal of the present study was to evaluate plasmatic sperm membrane protection and sperm quality parameters improvement in pure Spanish stallion sperm stored in conventional frozen straws, after thawing process, related to the addition of four different combinations of cholesterol (cholesterol of liver of pig 99%, SIGMA) and Equex-STM (Nova Chemical Sales, Scituate, MA, USA) in a lactose-EDTA-egg yolk home-made extender.

## 2. Materials and Methods

Semen was collected from six pure Spanish stallions aged 6 to 8 years belonging to a reproduction center in NE Spain (Equine Reproduction Center Torre Abejar, Garrapinillos, Zaragoza, Spain). Six ejaculates per stallion were collected from January to May 2004. Minimum quality was required to be frozen according to previous studies [2, 40, 41] (Table 1).

Semen was collected by artificial vagina, keeping the sample at 37.5°C in a double boiler immediately. Each ejaculate was diluted immediately after collection in glucose-EDTA extender [42] (1 : 1) and centrifuged to 400 g for 10 minutes to remove seminal plasma and possible gross contamination. The extender used for freezing/thawing semen consisted of lactose-EDTA-egg yolk [42] with cholesterol from pig spleen (Sigma C 3137–56) (125–150 mM) and Equex-STM (Paste 30 mg, Minitüb) (0.5–0.7%) added. Mixtures of 125 mM, 0.5% (I), 125 mM, 0.7% (II), 150 mM, 0.5% (III), and 150 mM, 0.7% (IV) were added to original Martin's extender (0 mM, 0.5%) (W) (Table 2).

After definitive dilution, samples were refrigerated at 5°C in a keeper box in the laboratory (Obstetric and Reproduction Area, Animal Pathology Department, Faculty of Veterinarian Sciences, University of Zaragoza, Spain). Then, equilibrated mixture was packed in 0.5 mL straws (100 × 10^6^ spz/mL). The straws were held at 4 cm above liquid nitrogen level for 10 minutes and then plunged into liquid nitrogen. Frozen samples were thawed by placing the straws at 37°C water bath for 21 seconds [43]. Three straws per treatment and boar were performed.

### 2.1. Sperm Evaluation

Total motility was measured by means of a computer-assisted semen analysis (CASA) system [44–46]. The following parameters were analysed: total motility (M), progressive motility (MP), and nonprogressive motility (NP). Sperm viability was evaluated by using eosin-nigrosin stain as described in [47]. A semen sample was diluted 1 : 1 with stain solution (5% eosin, 10% nigrosin in a citrate solution) and smeared. Live spermatozoa remained unstained. The percentage of live spermatozoa was expressed as viability. In addition, acrosomal integrity was evaluated under a phase contrast microscope after a 1 : 1 dilution in buffered 8% glutaraldehyde solution [48]. The percentage of spermatozoa with intact acrosome was determined. Membrane functional integrity was further assessed by the hypoosmotic swelling test (HOST) [49]. The technique consisted of incubating 30 *μ*L of diluted semen with 100 *μ*L of BTS hypoosmotic solution (100 mOsm/Kg) at 37°C for 15 min. The samples were then fixed in 8% glutaraldehyde buffered solution. The proportion of spermatozoa with swollen or coiled tails was considered as HOST positive as well as the integrity of acrosome after hypoosmotic incubation to determine the osmotic resistance (ORT). To evaluate spermatozoa capacitation state, chlortetracycline (CTC) fluorescence assay was performed [50]. Sperm samples were thawed at 37°C for 21 s and resuspended in PBS. Sperm-PBS was mixed with CTC solution (1 : 1), incubated at 37°C for 30 seconds, and subsequently fixed with glutaraldehyde (1 : 2). Briefly, the CTC solution was composed of 5 *µ*L CTC, 5 *µ*L cysteine, and 40 *µ*L buffer (20 mmol/L TRIS-HCl and 130 mmol/L NaCl), kept at 37°C, and incubated for 30 min. One hundred cells were assessed for each preparation under a differential interference microscope equipped with epifluorescence (excitation 450~490 nm: B2-A filter, 400x). Capacitated sperm (FL) consisted of low fluorescence in the postacrosomal and relatively bright fluorescence in the intact acrosome, almost no fluorescence over the entire head except a thin band of fluorescence in the equatorial segment, or low fluorescence in the postacrosomal and relatively bright fluorescence in the disintegrated acrosome.

### 2.2. Statistical Analysis

An analysis of factorial variance was applied (ANOVA) for the analysis, following the equation below, where the independent factors applied are stallion, ejaculate, and the different combinations of Equex-STM (percentage) and cholesterol (concentration) added to the different mixtures (called EQCOL factor) and the dependent variables are M, MP, NP, A, HOST, V, FL, and RT:

(1) δ=Stallioni+Ejaculatej+  Equex-STM-cholesterolk   +eijkm.

An interval of confidence of the 0.05% was applied in the model. Ejaculates for freezing/thawing fulfilling certain conditions were chosen (Table 1). Ejaculates data have been incorporated into the ANOVA in order to compare the variations of the factors implied (stallion, extraction, and the EQCOL) in respect of sperm quality. All the factors tested were independent of each other. Variables have been consistent with respect to the presence or absence of missing values. Tukey's Studentized Range (HSD) test has been used in order to establish significant mean comparisons between the mixtures of EQCOL. Statistical analysis was performed using the SPSS 11.0 Windows Package.

## 3. Results

We identified interactions between extender and stallion in all the parameters analysed (M, MP, NP, HOST, V, FL, and RT), indicating the importance of individual factor and ejaculate. Interactions of the extender with the fixed factor extraction in M, MP, NP, HOST, and A were obtained.

No interactions between the extender and the fixed factor *EQCOL* in any dependent variables, indicating that there is not different behavior of the extender combinations depending on the different combinations of Equex-STM (percentage) and cholesterol (concentration) added (Table 3). However, closed proximity to significance observed for FL in this fixed factor motivated us to analyse the different implications of each extender combination in this apparently annoying result (by Tukey's Studentized Range test). That is the reason why significant differences in the mean comparison of the extender combinations were identified for A (IV > II > I), HOST (IV > I), and FL (I > II > IV; W > II) (Table 4) after thawing process.

## 4. Discussion

Cryopreservation and thawing of equine sperm led to a significant decrease in the number of CASA-measured motile sperms to 11.3 ± 5.8% in May [45], which does not exactly correspond with the obtained results (6.78 ± 10.319). Moreover, CASA-measured objective motility and sperm survival rate after thawing process showed a marked lack of significant effect related to the different combinations of Equex-STM-cholesterol. However, sperm subjective motion capacity (analyzed by researchers) was modified by the different combinations of Equex-STM-cholesterol (data not shown), which indicates that it is not a good sperm quality parameter to check in other related experiments, such as was observed by different authors reviews, [8, 9, 14, 16].

As far as the authors know, first descriptive results in the literature of seminal quality of postthawed pure Spanish breed sperm are showed (Table 5).

Stallions were selected for this study following the criteria of the different authors reviews which found bad cryosurvival sperm results in those that failed in diverse seminal cooling studies [43, 44, 52]. This preliminary cooling sperm study was made by Gil et al. (2005, data not published), discarding those stallions which presented sperm quality deficiencies in the cooling process such as recommend Aurich [16]. The obtained results have demonstrated the significance of individual (M, MP, NP, HOST, V, FL, and RT) and extraction (M, MP, NP, HOST, and A), indicating the different behavior of the extender combinations depending on the horse employed or collection moment, as observed by other authors [19, 45, 51, 53–56].

Low density lipoproteins extracted from hen egg yolk have been also tested as cryoprotective media with an optimum extender concentration of 8%, in order to protect the spermatozoa against cold shock due to its protective effect in membranes [26]. On the other hand, it has been suggested that a close relationship among cold susceptibility, lipid phase transition, and lipids profile (polyunsaturated/saturated fatty acids) in gametes of ram, fowl, and bee spermatozoa exists [57] but no studies are carried out in stallions. We identified no interactions of the extender with the fixed factor *EQCOL* in any dependent variables, indicating that there is no different behaviors of the extender combinations depending on the laboratory technique used. However, significant differences in the mean comparison of the extender combinations were identified for A (IV > II > I), HOST (IV > I), and FL (I > II > IV; W > II) (Table 4) after thawing process in a posterior statistical analysis (Turkey's Studentized Range test). This result confirms that the combination of Equex-STM (0.5%) mixed with cholesterol (125 mM) has obtained better sperm quality results than those of original Martin's extender, possibly due to better balance on lipids membrane profile and its positive relationships with oxidative stress caused by ROS.

The function of the Equex-STM is related to outer cleaning of the sperm plasmatic membrane, at the time of the separation of the plasmatic and spermatic fractions of the ejaculate [38, 58]. Different authors reviews [38, 40, 43, 59] have found significant differences by using different percentages of Equex which contradicts the results obtained in this study, with no significant differences in sperm quality in neither of the two percentages used. We hypothesize that all the effects observed in the results were related to cholesterol adding (125 mM), independently of Equex adding (0.5 or 0.7%).

After analysing by fluorescence trials, we have concluded that the extender composed of 0.5% of Equex-STM and 125 mM of cholesterol has improved stallion semen quality after thawing than original Martin's extender.

## Figures and Tables

**Table 1:** Means and minimum sperm quality parameters for ejaculates.

Statistics	Concentration (Spr/mL)	Motility (%)	Viability (%)	Intact acrosome (%)	HOS test (%)
*N *	36	36	36	36	36
Mean	266,86	74,44	65,69	62,36	58,22
SD	8890,18	73,96	235,18	85,15	168,92
CV	94,28	8,60	15,33	9,227	12,99

Minimum required	>200	>60%	>50%	>50%	>50%

Spr/mL: millions of spermatozoa per milliliter. %: percentage of positive evaluated spermatozoa. *N*: observation data. SD: standard variation. CV: coefficient of variation. HOS test: hyperosmotic test.

**Table 2:** Composition of the Equex-STM-cholesterol mixtures tested.

Mixture	Cholesterol (mM)	Equex-STM (%)
White	0	0.5
I	125	0.5
II	125	0.7
III	150	0.5
IV	150	0.7

m/M: millimolar. %: percentage in total volume.

**Table 3:** General linear model procedure for independent variable (*t*).

Dependent variable	*n*	*R* ^2^	CV	Root MSE	Mean	Independent variable (Pr > *F*)		
						Stallion	Ejaculate	EQCOL
M	396	0.68	28.57	13.72	48.03	<0.0001	<0.0001	0.9850
MP	210	0.49	94.53	14.66	15.51	<0.0001	<0.0001	0.5153
NP	210	0.20	136.4	16.87	12.37	0.0009	0.0006	0.1798
A	396	0.45	26.63	12.49	46.89	<0.0001	<0.0001	0.1691
HOST	396	0.38	27.30	11.67	42.76	<0.0001	<0.0001	0.0811
V	384	0.56	28.50	13.97	49.02	<0.0001	0.2194	0.3268
FL	61	0.47	68.23	8.01	11.74	0.0009	0.0413	0.0160
RT	121	0.35	38.57	8.80	22.81	<0.0001	0.0772	0.6244

M: individual motility; MP: progressive motility; NP: nonprogressive motility; A: acrosome integrity; HOST: hypoosmotic swelling test; FL: fluorescence test; RT: resistance test; *n*: observation data; *R* ^2^: regression coefficient; CV: coefficient of variation; Root MSE: root of mean standard error; EQCOL: tested Equex-STM-cholesterol mixtures.

**Table 4:** Tukey's Studentized Range (HSD) test for EQCOL.

EQCOL comparison		Difference between means	95% confidence limits	
Acrosome integrity				
IV	I	4.125	0.033	8.217
IV	II	4.722	0.639	8.814
HOS test				
IV	I	4.972	1.146	8.798
Fluorescence				
I	IV	8.026	1.583	14.468
I	II	11.083	4.513	17.653
W	II	7.333	0.763	13.093

Means with nonsignificant comparisons have not been added into the table (Alpha = 0.05). W: 0–0.5; I: 125–0.5; II: 125–0.7; III: 150–0.5; IV: 150–0.7 (cholesterol mM-Equex-STM%). HOS: hypoosmotic swelling test.

**Table 5:** Descriptive statistics for sperm quality parameters after thawing.

Statistics	M	MP	NP	A	HOST	FL	RT
*N *	180	100	100	180	180	60	120
Mean	28.139	6.7800	10.670	36.600	35.628	11.733	22.750
SD	13.496	10.319	20.142	13.648	12.302	9.9930	10.348
CV	47.961	152.19	188.78	37.290	34.529	85.168	45.485

M: individual motility; MP: progressive motility; NP: nonprogressive motility; A: acrosome integrity; HOST: hypoosmotic swelling test; FL: fluorescence test; RT: resistance test; *N*: observation data; SD: standard variation; CV: coefficient of variation.

## References

[1] Vishwanath R (2003). Artificial insemination: the state of the art. *Theriogenology*.

[2] Blanchard TL, Varner DD (2002). *Manual of Equine Reproduction*.

[3] Adams SL, Hessian PA, Mladenov PV (2004). Cryopreservation of sea urchin (Evechinus chloroticus) sperm. *Cryo-Letters*.

[4] Drokin SI, Stein H, Govorukha TP (2003). Ultrastructure of carp Cyprinus carpio spermatozoa after cooling, dilution and freeze-thawing. *Cryo-Letters*.

[5] Gwo J-C, Wu C-Y, Chang W-SP, Cheng H-Y (2003). Evaluation of damage in Pacific oyster (Crassostrea gigas) spermatozoa before and after cryopreservation using comet assay. *Cryo-Letters*.

[6] Fernández-Santos MR, Esteso MC, Soler AJ, Montoro V, Garde JJ (2005). The effects of different cryoprotectants and the temperature of addition on the survival of red deer epididymal spermatozoa. *Cryo-Letters*.

[7] Grischenko VI, Dunaevskaya AV, Babenko VI (2003). Cryopreservation of human sperm using rapid cooling rates. *Cryo-Letters*.

[8] Medeiros ASL, Gomes GM, Carmo MT, Papa FO, Alvarenga MA (2002). Cryopreservation of stallion sperm using different amides. *Theriogenology*.

[9] Medeiros CMO, Forell F, Oliveira ATD, Rodrigues JL (2002). Current status of sperm cryopreservation: why isn’t it better?. *Theriogenology*.

[10] Weitze KF, Petzoldt R (1992). Preservation of semen. *Animal Reproduction Science*.

[11] Samper JC, Morris CA (1998). Current methods for stallion semen cryopreservation: a survey. *Theriogenology*.

[12] Samper JC (2001). Management and fertility of mares bred with frozen semen. *Animal Reproduction Science*.

[13] Saragusty J, Gacitua H, Pettit MT, Arav A (2007). Directional freezing of equine semen in large volumes. *Reproduction in Domestic Animals*.

[14] Clulow JR, Mansfield LJ, Morris LHA, Evans G, Maxwell WMC (2008). A comparison between freezing methods for the cryopreservation of stallion spermatozoa. *Animal Reproduction Science*.

[15] Sieme H, Martinsson G, Rauterberg H (2003). Application of techniques for sperm selection in fresh and frozen-thawed stallion semen. *Reproduction in Domestic Animals*.

[16] Aurich C (2008). Recent advances in cooled-semen technology. *Animal Reproduction Science*.

[17] Troedsson MHT, Liu IKM, Crabo BG (1998). Sperm transport and survival in the mare. *Theriogenology*.

[18] Sieme H, Harrison RAP, Petrunkina AM (2008). Cryobiological determinants of frozen semen quality, with special reference to stallion. *Animal Reproduction Science*.

[19] Loomis PR, Graham JK (2008). Commercial semen freezing: individual male variation in cryosurvival and the response of stallion sperm to customized freezing protocols. *Animal Reproduction Science*.

[20] Bedford SJ, Varner DD Cryopreservation induces capacitation-like changes in acrosomal status of stallion spermatozoa..

[21] Baumber J, Ball BA, Gravance CG, Medina V, Davies-Morel MCG (2000). The effect of reactive oxygen species on equine sperm motility, viability, acrosomal integrity, mitochondrial membrane potential, and membrane lipid peroxidation. *Journal of Andrology*.

[22] Combes GB, Varner DD, Schroeder F, Burghardt RC, Blanchard TL (2000). Effect of cholesterol on the motility and plasma membrane integrity of frozen equine spermatozoa after thawing. *Journal of Reproduction and Fertility. Supplement*.

[23] Neild DM, Gadella BM, Chaves MG, Miragaya MH, Colenbrander B, Agüero A (2003). Membrane changes during different stages of a freeze-thaw protocol for equine semen cryopreservation. *Theriogenology*.

[24] Heitland A (1995). Motility and fertility of stallion spermatozoa cooled and frozen in a modified skim milk extender containing egg yolk and liposome. *Equine Reproduction VI*.

[25] Zahn FS, Papa FO, Dell’ Aqua JA (2002). Cholesterol incorporation on equine sperm membrane: effects on post-thaw sperm parameters and fertility. *Theriogenology*.

[26] Moussa M, Martinet V, Trimeche A, Tainturier D, Anton M (2002). Low density lipoproteins extracted from hen egg yolk by an easy method: cryoprotective effect on frozen-thawed bull semen. *Theriogenology*.

[27] Peña A, Linde-Forsberg C (2000). Effects of equex, one- or two-step dilution, and two freezing and thawing rates on post-thaw survival of dog spermatozoa. *Theriogenology*.

[28] Tsutsui T, Hase M, Hori T (2000). Effect of addition of Orvus ES paste to frozen canine semen extender on sperm acrosomes. *Journal of Veterinary Medical Science*.

[29] Tsutsui T, Hase M, Tanaka A, Fujimura N, Hori T, Kawakami E (2000). Intrauterine and intravaginal insemination with frozen canine semen using an extender consisting of orvus ES paste-supplemented egg yolk tris-fructose citrate. *Journal of Veterinary Medical Science*.

[30] Nizański W, Dubiel A, Bielas W, Dejneka GJ (2001). Effects of three cryopreservation methods and two semen extenders on the quality of dog semen after thawing. *Journal of Reproduction and Fertility. Supplement*.

[31] Peña AI, López-Lugilde L, Barrio M, Becerra JJ, Quintela LA, Herradón PG (2003). Studies on the intracellular Ca^2+^ concentration of frozen-thawed dog spermatozoa: influence of equex from different sources, two thawing diluents and post-thaw incubation in capacitating conditions. *Reproduction in Domestic Animals*.

[32] Tsutsui T, Hase M, Hori T, Ito T, Kawakami E (2000). Effects of orvus ES paste on canine spermatozoal longevity after freezing and thawing. *Journal of Veterinary Medical Science*.

[33] Cochran JD (1984). *Effects of Centrifugation, Glycerol Level, Cooling to 5°C, Freezing Rate on the Post-Thaw Motility of Equine Spermatozoa*.

[34] Von Baer L, Helleman C (1999). Cryopreservation of Llama (Lama glama) Semen. *Reproduction in Domestic Animals*.

[35] Pursel VG, Schulman LL, Johnson LA (1978). Effect of Orvus ES Paste on acrosome morphology, motility and fertilizing capacity of frozen-thawed boar sperm. *Journal of animal science*.

[36] Abaigar T, Holt WV, Harrison RAP, Del Barrio G (1999). Sperm subpopulations in Boar (Sus scrofa) and Gazelle (Gazella dama mhorr) semen as revealed by pattern analysis of computer-assisted motility assessments. *Biology of Reproduction*.

[37] El-Alamy MA, Foote RH (2001). Freezability of spermatozoa from Finn and Dorset rams in multiple semen extenders. *Animal Reproduction Science*.

[38] Akourki A, Gil L, Echegaray A (2004). Effect of the extender supplement Equex-STM on cryopreserved semen in the Assaf sheep. *Cryo-Letters*.

[39] Thomas PGA, Surman V Addition of sodium dodecyl sulphate to tris-citrate extender improves motility and longevity of frozen-thawed canine spermatozoa.

[40] Amann RP, Pickett BW (1984). *An Overview of Frozen Equine Semen: Procedures for Thawing and Insemination of Frozen Equine Spermatozoa*.

[41] Juhász J, Nagy P, Kulcsár M, Huszenicza G (2000). Methods for semen and endocrinological evaluation of the stallion: a review. *Acta Veterinaria Brno*.

[42] Martin JC, Klug E, Günzel AR (1979). Centrifugation of stallion semen and its storage in large volume straws. *Journal of Reproduction and Fertility. Supplement*.

[43] Amann RP, Pickett BW Principles of cryopreservation and a review of cryopreservation of stallion spermatozoa.

[44] Verstegen J, Iguer-Ouada M, Onclin K (2002). Computer assisted semen analyzers in andrology research and veterinary practice. *Theriogenology*.

[45] Warnke C, Tuchscherer A, Alm H, Kanitz W, Blottner S, Torner H (2003). Characterisation of movement pattern and velocities of stallion spermatozoa depending on donor, season and cryopreservation. *Acta Veterinaria Hungarica*.

[46] Bruemmer JE (2006). Collection and freezing of epididymal stallion sperm. *Veterinary Clinics of North America: Equine Practice*.

[47] Eliasson R, Treichl L (1971). Supravital staining of human spermatozoa. *Fertility and Sterility*.

[48] Pursel VG, Johnson LA (1974). Glutaraldehyde fixation of boar spermatozoa for acrosome evaluation. *Theriogenology*.

[49] Jeyendran RS, Zaneveld LJ (1986). Human sperm hypoosmotic swelling test. *Fertility and Sterility*.

[50] Dubé C, Beaulieu M, Reyes-Moreno C, Guillemette C, Bailey JL (2004). Boar sperm storage capacity of BTS and Androhep Plus: viability, motility, capacitation, and tyrosine phosphorylation. *Theriogenology*.

[51] Metcalf ES (2007). The efficient use of equine cryopreserved semen. *Theriogenology*.

[52] Arruda RP, Ball BA, Gravance CG, Garcia AR, Liu IKM (2002). Effects of extenders and cryoprotectants on stallion sperm head morphometry. *Theriogenology*.

[53] Ball BA, Medina V, Gravance CG, Baumber J (2001). Effect of antioxidants on preservation of motility, viability and acrosomal integrity of equine spermatozoa during storage at 5°C. *Theriogenology*.

[54] Thurston LM, Watson PF, Holt WV (2002). Semen cryopreservation: a genetic explanation for species and individual variation?. *Cryo-Letters*.

[55] Loomis PR (2006). Advanced Methods for Handling and Preparation of Stallion Semen. *Veterinary Clinics of North America: Equine Practice*.

[56] López-Fernández C, Crespo F, Arroyo F (2007). Dynamics of sperm DNA fragmentation in domestic animals. II. The stallion. *Theriogenology*.

[57] Arav A, Pearl M, Zeron Y (2000). Does lipid profile explain chilling sensitivity and membrane lipid phase transition of spermatozoa and oocytes?. *Cryo-Letters*.

[58] Vidament M, Vincent P, Yvon JM, Bruneau B, Martin FX (2005). Glycerol in semen extender is a limiting factor in the fertility in asine and equine species. *Animal Reproduction Science*.

[59] Henry M, Snoeck PPN, Cottorello ACP (2002). Post-thaw spermatozoa plasma membrane integrity and motility of stallion semen frozen with different cryoprotectants. *Theriogenology*.

